# Fiber‐enriched botanicals: A therapeutic tool against certain metabolic ailments

**DOI:** 10.1002/fsn3.2920

**Published:** 2022-08-26

**Authors:** Waseem Khalid, Muhammad Sajid Arshad, Ayesha Jabeen, Faqir Muhammad Anjum, Tahira Batool Qaisrani, Hafiz Ansar Rasul Suleria

**Affiliations:** ^1^ Department of Food Science Faculty of Life Sciences Government College University Faisalabad Pakistan; ^2^ 98873 University of the Gambia Banjul The Gambia; ^3^ IFANCA Halal Apex (Pvt.) Limited Faisalabad Pakistan; ^4^ Department of Agricultural Engineering and Technology Ghazi University Dera Ghazi Khan Pakistan; ^5^ Department of Agriculture and Food Systems The University of Melbourne Melbourne Australia

**Keywords:** COVID‐19, diseases, fiber, immune system, plant‐based foods

## Abstract

Plant‐based foods are natural sources including vegetables, fruits, cereals and legumes. These foods consist of various types of nutrients in which carbohydrate is the basic component. However, some plant‐based diets contain carbohydrates in the form of fiber. The fiber is usually a nondigestible polysaccharide that is not digested in the human body. It is present in the form of soluble or insoluble in different part of foods like peel, bran, pulp and grain. Pectin, beta‐glucan, mucilage, psyllium, resistant starch and inulin are soluble fiber, and cellulose, hemicellulose and lignin are insoluble fiber attained from plant foods. The major function enhances immunity by creating gastrointestinal barrier, mucus production, immune cell activity and IgA level. Previous evidences showed that peoples with strong immunity have fewer chances of viral disease. A recent viral disease named COVID‐19 spread in the world and millions of peoples died due to this viral disease. Coronavirus mostly attacks humans that suffer with weak immune system. It is due chronic diseases like diabetes and CVD (cardiovascular disease). The current review shows that fiber‐containing plant‐based foods boost immunity and aid human against COVID‐19. The therapeutic role of fiber in the human body is to control the risk of hypertension and diabetes because a high‐fiber diet has the ability to lower cholesterol, blood pressure and blood sugar. Fibers aid in GIT (gastrointestinal tract) and prevent constipation because it absorbs water and adds bulk to stool.

## Graphical abstract

The current review shows that fiber‐containing plant‐based foods boost immunity and aid human against covid‐19. The therapeutic role of fiber in the human body; is controlled the risk of hypertension and diabetes because a high‐fiber diet is the ability to lower cholesterol, blood pressure and blood sugar. Its aid in GIT and preventing constipation because it absorbs water and adds bulk to stool.

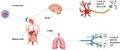

## 1 INTRODUCTION

### 1.1 Overview of fiber

Fiber, which is also called roughage. It is a component of plant‐based foods (grains, nuts, beans, vegetables and fruits) which are not digested by our body (Thilagavathi et al., 2020). This nondigestible part passed out from the body without any digestion process. As such, it keeps the digestive system healthy and clean, helps with the movement of the bowels and flushes cholesterol and other harmful substances out of the body (Kandasamy & Shanmugapriya, 2015).

Fiber is a carbohydrate that is not digested in the human digestive system (Mudgil & Barak, 2013). Whole fruits, beans, whole grains and vegetables are a great source of fiber (Erkkilä et al., 2005). The plant‐based food sources of fiber and their types are presented in Table 1. Most carbohydrates are broken down into small sugar molecules (Meier et al., 2017). But the fiber cannot be broken into simple molecules and passes out of the body in undigested form (Buttriss & Stokes, 2008). Fiber aids in regulating the glycemic index in humans and helps to maintain hunger and blood sugar balance (Brownlee, 2011).

**TABLE 1:** Types and plant‐based food sources of fiber

Fiber	Type (soluble or insoluble)	Plant‐based food sources	References
Pectin	Soluble	Pears, apples, guavas, quince, plums, gooseberries, oranges and other citrus fruits contain large amounts of pectin, while soft fruits, like cherries, grapes and strawberries, contain small amounts of pectin	(Dranca & Oroian, 2018)
Mucilage	Soluble	Flaxseeds, chia seeds, *Aloe vera*, okra and figs	(Muñoz et al., 2012)
Beta‐glucan	Soluble	Barley, oats and whole grains	(Kaur et al., 2020)
Psyllium	Soluble	Psyllium husk	(Khalid et al., 2021; Qaisrani et al. 2014)
Resistant Starch	soluble	Oats, rice, grains, beans, legumes, potato, green bananas and maize	(Fuentes‐Zaragoza et al., 2010)
Inulin	soluble	Onions, wheat, garlic, Jerusalem artichoke, chicory, root, wheat and barley	(Murphy, 2001; Roberfroid, 1993, 2005, 2007)
Cellulose	Insoluble	Bran, legumes, nuts, peas, roots, cabbage, apple skins, pineapple and banana leaves, sugarcane rind and coconut husks	Reddy & Yang, 2005)
Hemicellulose	Insoluble	Bran, nuts, legumes and whole grains	(Kalyani Nair et al., 2010)
Lignin	Insoluble	Pasta, rice, cereal, citrus fruits, whole grain and oat	(Mongeau & Brooks, 2001)

Dietary fiber is a plant‐based nutrient that is also called bulk. It is a carbohydrate but unique from other carbohydrates. It is nondigestible, but some of the components are partially digestible. Therefore, it passes through the intestinal tract without any change (Soliman, 2019).

Dietary fiber is classified by origin, chemical composition and physicochemical properties. Along with that, it is subcategorized by the degree of polymerization, like chain length. Predominantly, all the properties of dietary fiber can impact fermentation by microbes (Mei et al., 2010). Plant‐based fibers can be derived from fruits, vegetables, cereals, grains, legumes and nuts. The fiber originated from plants has varied chemical and physiochemical properties (McRorie & Fahey, 2013). The banana is a fruit that contains inulin which is a fructan and resistant starch, while the apple is rich in pectin. Diet having plant‐based food is rich in different types of dietary fiber and provides the most diverse composition of the microbiota (Jha et al., 2019; Judprasong et al., 2011). Physiochemical characteristics, including solubility, viscosity and fermentability, influence the therapeutic effects of consumption along with fermentation (Schieber et al., 2001).

The lower retention of glucose and bile corrosive coupling is the physiological advantage of psyllium, gelatin and beta‐glucan, which aid the growth of microbiota in the gastrointestinal tract. Polydextrose and soluble corn fiber are soluble fibers that are poorly digested by human alimentary enzymes (Holscher et al., 2015). Fructan is an inulin type‐fiber which is present in artichokes, bananas, garlic, agave, asparagus, leeks, wheat and onions (Holscher et al., 2014).

The level of polymerization of fructans are affected the maturation profile in people. Proof of physiological advantages of fructans in clinical investigations is restricted. The investigation of rodents has exhibited that the utilization of inulin‐type strands differentially reduces body weight, convergence of blood glucose and blood cholesterol (Rendón‐Huerta et al., 2012).

According to several studies, higher consumption of dietary fiber is beneficial for a variety of health outcomes, while many results showed that there has been no attempt to systematically capture the breadth of outcomes associated with intake of dietary fiber or assess the quality and strength of evidence on the association of dietary fiber intake, different medical conditions and health outcomes (Veronese et al., 2018). Generally, the fiber in food (fermented fiber) is the basis of a healthy diet. While prebiotics are specialized food ingredients that affect the final fermentation of products. It is beneficial for the host's health and specific bacteria (Platta, 2020). The study of rodents has demonstrated that the consumption of inulin‐type fibers differentially reduces body weight and the concentration of blood glucose and blood cholesterol (Rendón‐Huerta et al., 2012).

Holscher (2017) demonstrated the effect of prebiotics and fiber utilization on the metabolic capacity and composition of human gastrointestinal microbiota. The impacts of physiochemical properties of complex sugar can improved the health of human microbiota.

#### 1.1.1 Soluble fiber in plant‐based foods

Soluble fiber is present in barley, nuts, beans, seeds, peas, lentils, fruits, vegetables and oat bran. This is also present in psyllium, which is a fiber supplement. This fiber is generally derived from the inner flesh of foods that are plant‐based (Mudgil, 2017). The soluble fiber is mainly associated with the pulp or flesh of foods like oranges and potatoes (Lachman et al., 2005). Fructan is a type of inulin that is found in artichokes, bananas, garlic, agave, asparagus, leeks, wheat and onions (Holscher et al., 2014). The soluble fiber attracts water and gets converted into a gel by digestion. These fibers dissolve in water and form a viscous and gelatinous structure in the human digestive system (Wang et al., 2021).

Soluble fibers can change many metabolic and physiological processes involved in lowering cholesterol levels. These included stomach emptying, carbohydrate and fat digestion and absorption, nutrient intake, postprandial blood response, fermentation in the colon, liver metabolism and fecal excretion (Rideout et al., 2008).

Soluble dietary fibers are present in all food products separately or in combination with insoluble dietary fiber in small amounts, which contribute towards dietary fiber. Soluble dietary fibers are associated with a significant role in human physiological function. The potential benefits of soluble fibers are including a reduction in blood pressure and cholesterol levels, the prevention of gastrointestinal problems, protection against several types of cancer (including prostate, breast and prostate cancer) and increased bioavailability of minerals (Chawla & Patil, 2010).

Pectin is a soluble fiber which is present in jellies and jams, mixed with partially digested foods in the same way as gum, inulin, beta‐glucan and mucilage (Yanniotis et al., 2007). The consumption of viscous forming and water‐soluble fibers, including pectin and psyllium reduces the cholesterol level in human plasma (Butt et al., 2007).

Psyllium is included in nonfermentable fiber, while its greater viscosity and solubility cause unique therapeutic effects including reduction in blood cholesterol levels and improved glycemic control (Holscher et al., 2015).

The beta‐glucan and pectin are highly fermentable along with high viscosity and solubility (Schieber et al., 2001). These fibers are found in whole grains including barley and oats (beta‐glucan) and fruits like apples (pectin) (McRorie & Fahey, 2013). Reduced absorption of glucose and bile acid‐binding are the physiological benefits of psyllium, pectin and beta‐glucan which strongly impact the microbiota of the gastrointestine. The soluble and nonviscous fibers are fermented by gastrointestinal microbiota like resistant maltodextrins, resistant starch, inulin, soluble corn fiber and ply‐dextrose (Holscher et al., 2015).

The soluble fiber improves a healthy heart by modulating the cholesterol level in the body and lowering blood pressure. For example, pectin helps to reduce fat absorption in the human body from different foods, while beta‐glucan is responsible for lowering the content of bad cholesterol. The soluble fiber lowers and regulates blood glucose levels in type 2 diabetic patients. Healthy blood glucose level leads to reduced inulin requirements in diabetics (Post et al., 2012).

#### 1.1.2 Insoluble fiber in plant‐based foods

The insoluble fiber cannot dissolve in water and remains intact when food moves through the gastrointestinal tract. Fiber is generally a part of plant‐based foods which is not digested and absorbed in the human body (McRorie & McKeown, 2017). Wheat brans, beans, nuts, whole wheat flour and vegetables including kale, potatoes, cauliflower and green beans are good sources of insoluble fiber.

Insoluble dietary fiber is abundant, inexpensive and rich in dietary fiber as a by‐product (Daou & Zhang, 2014). Advanced dietary fiber has been recently reported to promote heavy metal adsorption capacity by using chemical, physical and enzymatic methods (Feng et al., 2009; Park et al., 2013). The insoluble fiber is present in vegetables, whole grains and wheat bran. The insoluble fiber helps to cure constipation and complications like helping stool to pass out quickly through the intestine and bulking it up. Insoluble fiber helps to reduce the risk of colorectal cancer by speeding up the process of removal of waste from the digestive tract. The removal of harmful substances from the bloodstream of the human body depends upon the removal of water from the intestines along with stool. Due to lower loss of water from the body it is absorbed into the bloodstream again through intestinal walls (Huang et al., 2014).

The insoluble fiber aids in the passage of food from the body because it adds a bulky appearance to the stool and helps it pass quickly through the stomach and intestines, by increasing fecal bulk and helping the stool pass out easily (Forootan et al., 2018).

After entering the digestive system, insoluble fiber attracts water and sweeps waste materials from the large intestine. It is made up of cellulose and lignin, derived from the tough and outer skin of the plant. Generally, insoluble fiber is present in the skin of fruits and vegetables including pears, potatoes and apples. Foods packed with insoluble fiber generally have a chewy and tough texture. Insoluble fiber includes the skins of fruits and vegetables, the outer layer of cereal grains and wheat bran (Huang et al., 2014).

Cellulose is a dietary fiber that is poorly fermented by microbes present in the gut (Trompette et al., 2014). Insoluble fibers like cellulose are poorly fermented by microbes present in the gut. Their presence in the diet increases the transit rate of the gut and reduces the amount of time required for colonic bacterial fermentation of nondigested foodstuffs (McRorie, 2015).

## 2 FIBER‐RICH PLANT‐BASED FOODS

### 2.1 Fruits and their parts which are rich sources of fiber

The fruit is usually a sweet and fleshy product that contains pulp, skin and seeds. It is highly consumed in the world as food (Kumoro et al., 2020). These two types are fresh fruit and dried fruit, which are composed of different nutrients and many chemicals (Idah et al., 2007). Apples, pears, oranges, grapefruit, apricot, peach, plum, banana, mango, watermelon, melon, strawberries, raspberries, blueberries, kiwifruit, avocados, cherries, lemon, pineapple, pomegranate, papaya and guavas all contain both types of fiber.

Fruits are a rich source of soluble and insoluble fiber (Mcintosh & Miller, 2001). The fiber is present in the skin and pulp of fruits. The pectin, gum, mucilage, beta‐glucan and alginates are soluble fibers, and lignin or cellulose is an insoluble fiber attained from fruits (Bunzel et al., 2005).

The fruit ripening after harvesting causes three‐dimensional hydrated cell wall fiber components pectin that completely dissembled cellulose and hemicellulose. It allow access to microbes and enhance fermentation susceptibility (Low et al., 2015). The fruit is eaten and its digestion by the gastrointestinal tract breaks it into smaller particles and damages the cell walls of the fruit, which empowers superior enzymatic fermentation and breakdown of colonic bacteria. Some starchy fruits like plantain and banana also contained fiber from both cell wall and resistant starch stored within the cell (Garcia‐Mantrana et al., 2018). Basically, it is anticipated that the soluble fibers are completely fermented and equated quickly with the insoluble fibers, but freshly ripped fruits containing cell walls having insoluble fibers in a hybrid hydrated and moderately (Williams et al., 2017).

As per previous investigations, 100 g of dry apple contained about 46 g of insoluble fiber, 14 g of soluble fiber, 8–12 g of gelatin and fructooligosaccharide and 4.9% of complete dietary fiber correspondingly (Nawirska & Kwaśniewska, 2005). Similarly, banana contains around 43–49 g of dietary fiber, 1 g of inulin, 10–20 g of gelatin and 6 g of fructooligosaccharide in 100 g of dry matter (Emaga et al., 2007; Mohapatra et al., 2010). Indisputably, 100g of the husk of yellow enthusiasm organic product contains 20 g of gelatin and 60 g of complete insoluble dietary fiber (Yapo & Koffi, 2008). The level of polymerization of homogalacturonan in apple and banana strips and yellow enthusiasm natural product skin was depicted to be 78%, 67% and 93% individually (Yapo & Koffi, 2008).

The consumption of dietary fiber in the form of whole fruit has beneficial health effects including lowering LDL cholesterol, improving gut health, reducing cardiovascular diseases, reducing the risk of excessive weight gain, obesity, chronic heart diseases and type 2 diabetes, improving cancers and stroke (Veronese et al., 2018).

### 2.2 Different types of vegetables and their constituents rich sources of fiber

Vegetables are also like fruits, have low‐calorie fiber and can be consumed along with the skin of some fruits. Humans and animals consumed vegetables as food (Pennington & Fisher, 2009). The edible parts of plants are fruits, flowers, leaves, stems, seeds and roots. Brussels sprouts, artichokes, broccoli, collard greens, carrots, turnips, beets, celery root, Swiss chard, parsnips and kale are different types of vegetables (Khalid et al., 2021; Verkerk et al., 2009). Generally, the darker color of vegetables shows that they are rich in fiber content. Vegetables like beets, broccoli and carrots are rich in fiber. Artichokes contain the highest amount of fiber (a medium‐sized artichoke contains 10 g of fiber) (Villanueva‐Suárez et al., 2019).

Worldwide, potatoes are a staple vegetable and consumed largely. Potatoes are rich in starch and provide protein of high biological value which is not present in leafy vegetables (Camire et al., 2009). Whole potatoes are rich in potassium, vitamin C and dietary fiber (Khalid et al., 2020). According to several prospective studies, there is a relationship between potato consumption and health (Halton et al., 2006).

### 2.3 Different types of cereals and their constituents rich sources of fiber

Food products made with whole grain are rich in fiber. Cereals having wheat bran or oats are rich in fiber. On the other hand, cereals having rice or corn have less fiber. The whole grain cereals without excessive preservatives, flavoring, sugar and coloring showed more fiber than the highly processed cereals (Barrett et al., 2020). Oat bran is rich in soluble fiber which is beneficial for lowering the cholesterol level in the blood. Rice bran, wheat and corn are rich in insoluble fiber which is responsible for preventing constipation (Bernstein et al., 2013).

Oats and wheat are rich in fiber. Barley is a cereal that is often used in soups; 1 bowl of barley contains 6 g of fiber which is consumed in breakfast generally. Barley is rich in zinc which is a mineral that helps our body to build, repair and heal. Rice bran contains a low amount of dietary fiber up to 1.8–2.7 g per 100 g while oat bran contains 5.3–8.4 g per 100 g (Marlett et al., 1994).

Rice, wheat and oat have less hypolipidemic impact than some other grains because of less substance of dissolvable fiber. While rice‐wheat has 12%–23% of oil, a higher rate than other grain sources, oil has unsaponifiable issue fixation of up to 4.2% (Gerhardt et al., 1998). This γ‐oryzanol, β‐sitosterol, tocotrienols and unsaturated fats contribute to the decrease in cholesterol. These all contribute to the decrease in cholesterol (Berger et al., 2005).

Diet contain water‐dissolvable fiber that is played a basic partin decreasing the cholesterol level (Anderson et al., 1994). Oat gum, guar gum and gelatin are solvent strands which introduce hypocholesterolemic properties into humans (Cui & Roberts, 2009). As per an investigation, guar gum, oat wheat, gelatin, psyllium and insect bean gum reduce around 3%–20% of the cholesterol level (Anderson et al., 1994). The water‐dissolvable filaments do not have any effect on cholesterol levels (AL‐Rawi, 2007).

The capacity of different types of fiber to bind bile acids or bile salts is quite variable, and a high capacity seems to correspond with a hypocholesterolemic effect (Andersson & Hellstrand, 2012). This multiplies the union of bile salts, and catabolism of low‐thickness lipoprotein is improved and constrained by the low‐thickness lipoprotein receptors (Wood et al., 2007).

In vitro study suggested that bile acid‐binding by rice, oat, wheat, and corn brans is determined using a mixture of bile acids normally secreted in human bile. The result showed that cholesterol‐lowering by rice bran appears to be related to bile acid‐binding. Bile acid‐binding by oat bran (soluble fiber) may not be cholesterol‐lowering. Bile acid binding by wheat bran can prevent cancer and provide healthful properties (Kahlon & Chow, 2000).

### 2.4 Different types of legumes and their constituents rich sources of fiber

Legumes belong to a class of vegetables which also include peas, lentils and beans. These are among the most nutritious and versatile foods available globally (Graham & Vance, 2003). Peas, beans, lentils, peanuts, soybeans and chickpeas are some edible legumes. The types of legumes are differentiated on the basis of appearance, taste, use and nutrition. Legumes are generally used to describe the seeds of plants (Sánchez‐Chino et al., 2015). Legumes generally have no cholesterol, low fat, high folate, iron, magnesium and potassium (Messina, 2014).

Legumes contain soluble and insoluble fiber and beneficial fats. The legumes have insoluble fiber which slows down the digestion process and releases carbohydrates gradually, which tends to help keep the blood sugar level at its optimum level (Trinidad et al., 2010). Generally, seeds of legumes comprise additional dietary fiber than cereals and are a virtuous source of metabolically active soluble dietary fiber (Hughes & Swanson, 1989).

Navy beans are one of the richest sources of fiber. Black beans contain a large amount of magnesium and iron. Pinto beans are consumed as a staple food in the United States. The total dietary fiber content fluctuates from lentils 11.5% to guar 33.2%. Guar is an annoying source of soluble dietary fiber. The ratio of water‐soluble to insoluble fiber in legumes revealed that there is a higher percentage of insoluble fiber than soluble fiber. The foremost percentage of fiber in legumes comprises cellulose and hemicellulose, while a small proportion is of lignin and pectin. Guar seeds contain the highest amounts of lignin 2.0%, pectin 3.0% and cellulose 12.5%. Chickpeas are the best source of hemicellulose. The results of studies showed that chickpeas, field bean and guar contain more proportion of soluble fiber than other species of legumes, which are more nutritious and should be added to the diet (Khan et al., 2007).

## 3 IMMUNITY‐BOOSTING ROLE OF FIBER

Immunity is a complex biological system enriched in the ability to recognize and tolerate whatever belongs to the self and to perceive and dismiss. Immunity is the ability of multicellular living beings to oppose harmful microorganisms. Immunity has two types including innate and adaptive. Innate provides the first line of defense against infection whereas, adaptive provides a specific immune response directed at an invading pathogen. A plant‐based eating routine reinforces your resistant immune system to secure you against germs and microorganisms. A sound safe immune system is fundamental for diminishing the danger of pathogens since it can perceive transformations in cells (Klasing, 2007).

Plant‐based food sources expanded the intestinal valuable microbes which are useful and makeup 85% of the immune system (Arshad et al., 2020). The utilization of a lot of water, minerals like magnesium and zinc, micronutrients, spices, food plentiful in vitamins (C, D and E) and a better way of life can boost immunity against different diseases. The study suggested that glutathione is the most abundant molecular weight antioxidant. It is played a defensive role against oxidative damage to cells (Polonikov, 2020). The plant‐based nourishments play a fundamental part in improving the resistance of individuals to controlling COVID‐19 (Arshad et al., 2020).

A high fiber plant‐rich eating routine with a lot of natural products, vegetables and entire grains seems to help the development and support of valuable microorganisms. Certain accommodating organisms separate filaments into short‐chain unsaturated fats, which have been appeared to invigorate invulnerable cell movement (Randhawa et al., 2015).

Fiber assumes a significant part in stomach‐related wellbeing. Fiber is the fuel the colon cells use to keep them solid. Fiber likewise assists with keeping the stomach‐related plot streaming, by keeping your solid discharges delicate and normal. An eating regimen wealthy in fiber adds to the upkeep of a solid gut microbiota related to expanded variety and capacities (Hecht, 2004).

Soluble and insoluble fibers are found in various food sources, for example, vegetables, nuts, seeds, natural products and oats. Nonetheless, not a wide range of fibers are available in similar food classes. Fiber is found in dull food sources, for example, cereals, vegetables and tubers, and nondeveloped natural products like green bananas. While gelatins are more advanced in foods grown from the ground vegetables, though beta‐glucans and arabinoxylans are available in oats (Lovegrove et al., 2017). Although fibers are available in a wide range of plant‐based food sources but utilization is low in Western nations. The stronghold of nourishments with extricated or orchestrated nonedible starches or their utilization in dietary enhancements accordingly establishes a procedure to expand fiber admission. A wide range of these carb polymers and oligosaccharides are industrially accessible (Deehan & Walter, 2016). A portion of these mixtures are considered "prebiotics" for the reason that they provide medical advantages by specifically prompting advantageous bacterial populaces in the gut (Bindels et al., 2015). The task of just certain fibers as prebiotics dependent on these models is by one way or another subjective (Deehan et al., 2018). Basically, all filaments will initiate explicit moves in microbiota arrangement because of serious connections and compositional movements add to medical advantages (Bindels et al., 2015). Interestingly, the systems that have been shown to add to medical advantages don't depend on a specific usage of the starch but instead on metabolic mixtures (Koh et al., 2016), physiological changes (pH bringing down) or security of the bodily fluid layer (Schroeder et al., 2018; Zou et al., 2018). It could merit considering a move in the focal point of the prebiotic idea away from the particular impact of explicit dietary segments on gut microbial networks and rather toward environmental and useful outcomes of fiber maturation (Bindels et al., 2015).

**FIGURE 1:**
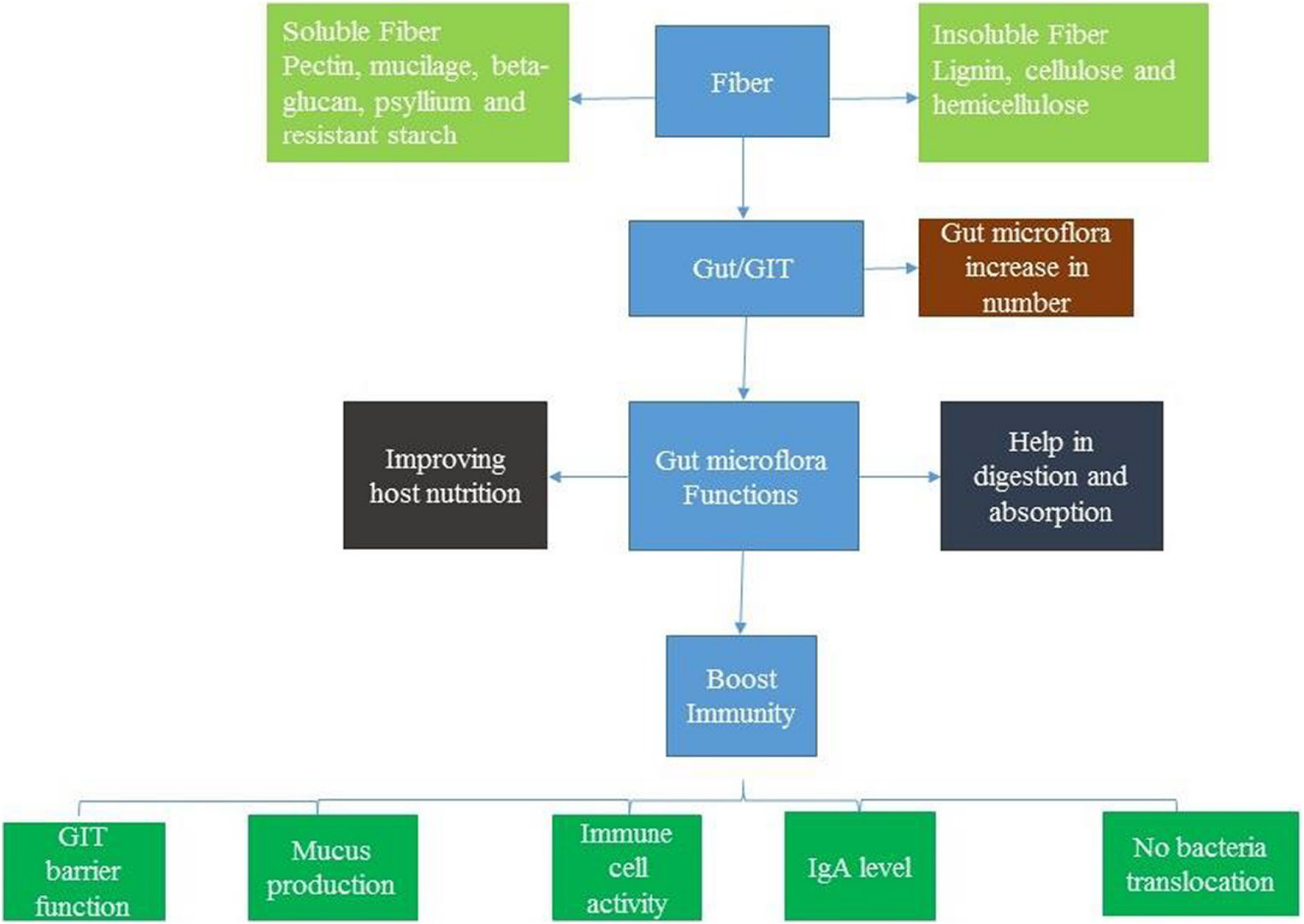
Plants derived fiber (soluble or insoluble) enter into GIT (gastrointestinal tract) and performed some functions. The major function enhances immunity by creating gastrointestinal barrier, mucus production, immune cell activity and IgA level

Fiber helps in digestion by enhancing bowel movement, supporting the immune system and providing food for the gut bacteria (Anderson et al., 2009). Bacteria present in our body also need food like living organisms to survive and fiber present in our daily diet is the food for these bacteria. The food for good bacteria is called prebiotics, which are present in fiber‐rich foods like garlic, onions and jackfruit. When the gut bacteria get fiber‐rich foods called prebiotics, then these bacteria enhance the immune system, digestive health and any other health benefits. The eating of fiber on daily base that can enhance gut microbe and perform their function properly to improve the immune system and gut health. The gastrointestinal tract is the principal immune organ in humans (Slavin, 2013). The lymphoid tissues accompanying with gut comprise almost 60% of lymphocytes present in the human body including Peyer's patches, nonaggregated and intracellular lymphocytes. Figure 1 represents the mechanism of boosting immunity by fiber‐rich plant foods.

The working of the immune system of the gut is influenced by the concentration of prebiotics in the diet which encourages the growth of health‐promoting bacteria in the colon (Roberfroid, 2007; Vos et al., 2007). Most probiotics are nondigestible carbohydrates that are fermented in the colon (Roberfroid, 2005). Inulin and other oligofructoses are the most studied dietary fiber. It can help to promote the growth of *bifidobacteria* in the colon. *Bifidobacteria* and *Lactobacilli* are the health‐promoting bacteria that generate short‐chain fatty acids and also stimulate the immune system (Vos et al., 2007). Many animal studies have shown that inulin has a favorable effect on the immune system and human studies also supported this hypothesis (Roberfroid, 2007). Oligofructose and inulin are not assimilated by pancreatic and brush border enzymes due to their movement inside the colon deprived of any restraint.

Inulin is fermented absolutely by the microbacteria in the colon which stimulates the progression of bifidobacteria. The fermentation results in the production of short‐chain fatty acids. The other fibers are also fermented in the gut to generate short‐chain fatty acids. The effects of bifidobacteria on non‐oligofructose fiber are not well defined yet. The health benifits of bifidobacteria are including depressing of intestinal pH to form acids after acclimatization of carbohydrates, protection from an intestinal infection, reduction of harmful bacteria, manufacturing of vitamins and minerals, bulking activity to inhibit constipation, inspiration of immune response, reduction of risks of colorectal cancer, assistance with indigestion and fascination with minerals exclusively calcium (Roberfroid, 2007; Vos et al., 2007).

The study was conducted in which indicate that the supplementation of inulin increases the bacterial content in feces and has an important effect on circulating lymphocytes (Watzl et al., 2005). Oligofructoses and inulin have been studied completely and have the beneficial effect of fermentable soluble fibers on gum arabic, oat beta‐glucan and others (Schley & Field, 2002).

The prebiotic fiber has a major role in the health and nutrition of infants which is generating great interest. The supplemented mixture of prebiotic fiber has various health benefits including a reduction in respiratory infections and atopic dermatitis, enhancing bowel function and promoting postnatal immune development (Veereman, 2007).

The potential of prebiotic fibers is to cure inflammatory bowel diseases. According to different studies on animals, it has been proved that prebiotic fiber reduces gut inflammation (Ewaschuk & Dieleman, 2006; Guarner, 2005). Recent study has indicated that prebiotic fibers reduce the risk of liver infection after having a liver transplant (Guarner, 2007). The previous human studies showed favorable responses of fibers against Cohn's disease, ulcerative colitis and pouchitis (Ewaschuk & Dieleman, 2006; Guarner, 2005). Pectin is a dietary fiber that has different structural characteristics. The structure of specific pectin influences the gastrointestinal immune barrier by interacting with immune cells or impacting intestinal microbiota directly. The pectin impact depends upon the structural characteristics of pectin including degree of esterification of methyl, acetylation, rhamnogalacturonan I and rhamnogalacturonan II neutral side chains (Prado et al., 2020).

The previous study reviewed that there is an interaction of the pectin structures with the immune barriers of gastrointestine. The effects of pectin include enhancing epithelial integrity, strengthening the mucus layer, inhibiting dendritic cell and macrophage response. The interaction between the immune barrier of the gastrointestinal and pectin is governed by recognition receptors included, Galectin 3, Toll‐like receptors 2 and 4. Additionally, some of the pectin can stimulate the abundance and diversity of beneficial microbial communities. The immune barriers of gastrointestine enhance the number of short‐chain fatty acids. Pectin enhances the intestinal immune barrier by inhibiting the adhesion of commensal bacteria and pathogens to the epithelial cells. Different studies illustrated that pectin is a powerful dietary fiber which prevents inflammatory conditions (Beukema et al., 2020).

## 4 FAVORABLE AGAINST DIFFERENT DISEASES

**FIGURE 2:**
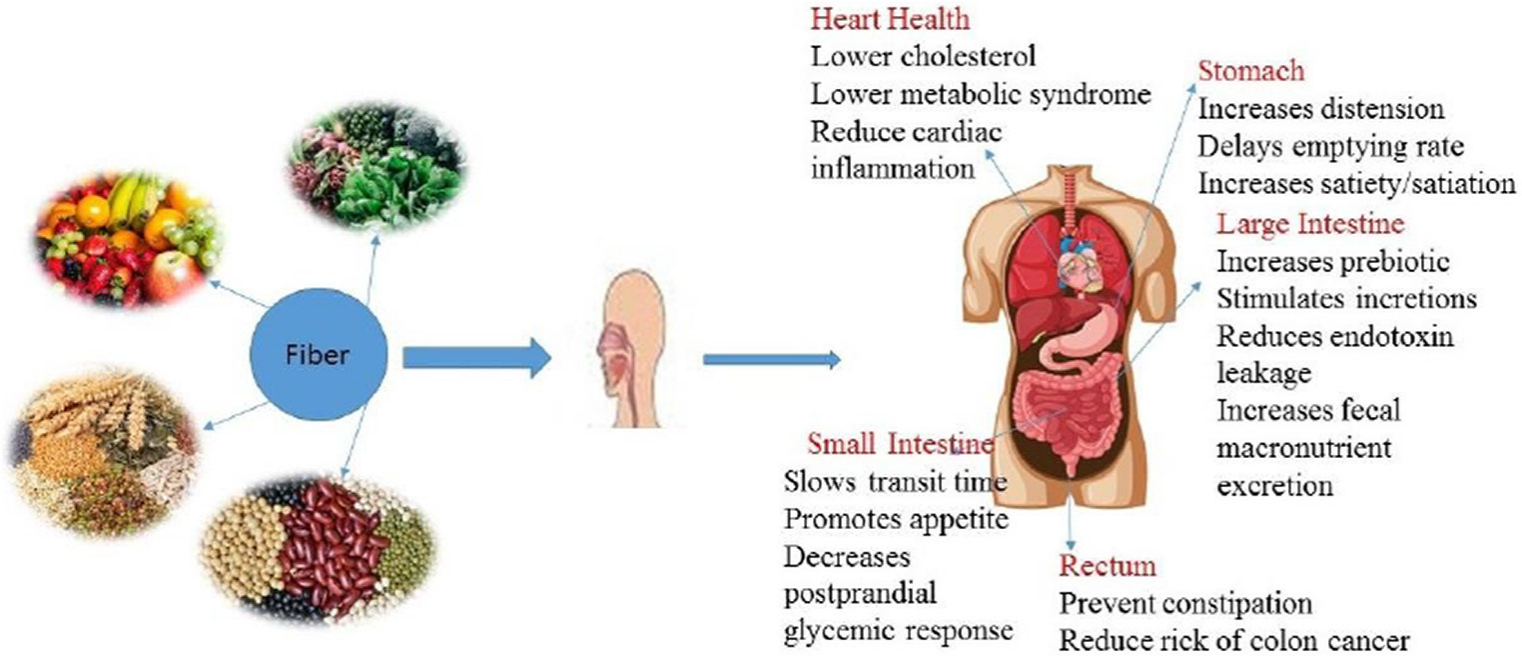
Major dietary sources of fiber and potential health benefits

Fiber is naturally present in vegetables, fruits, cereals and legumes. There are servals benefits to human health including heart health, stomach regulation, small or large intestine movement and preventing rectum cancer. Figure 2 shows the dietary sources of fiber and potential health benefits.

### 4.1 COVID‐19

Coronavirus disease (COVID‐19) is an emerging respiratory infection caused by the severe acute respiratory syndrome coronavirus (SARS‐CoV‐2), which has in recent times become a pandemic (Lai et al., 2020). Most COVID‐19 patients show mild to moderate symptoms, but about 15% of patients progress to severe pneumonia, and about 5% of patients eventually develop acute respiratory distress syndrome (ARDS), septic shock and multiple organ failure (Huang et al., 2020; Xu et al., 2020). Flu‐like symptoms of COVID‐19 usually appear 5–6 days after infection, and in some cases include cough, sore throat, fever, muscle and body aches and even loss of smell or taste **(**Galanakis, 2020 **)**.

**FIGURE 3:**
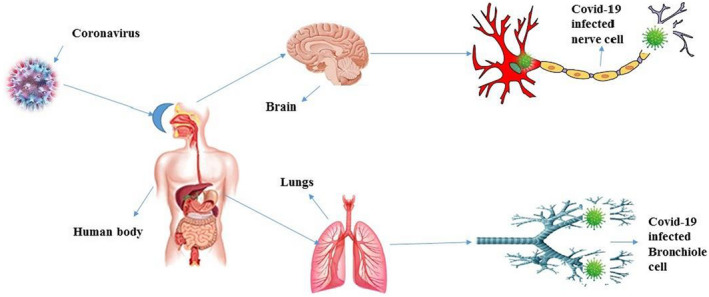
There are different entry routes of COVID‐19 in to the body. Corona viruses enter into the body through the nose, mouth and eyes. Viruses enter into brain and lungs and attack nerve and bronchiole cells. However, the virus damages the cells and creates a health problem

In humans, coronaviruses are included in the spectrum of viruses that cause the common cold and the recent severe acute respiratory syndrome. Emerging infectious diseases such as SARS‐CoV‐2 pose a major threat to public health. The new coronavirus has spread speedily to many countries and has been declared a pandemic by the World Health Organization. It usually causes the virus infection of COVID‐19. It is very likely that people with low immune response are infected (Arshad et al., 2020). The different entry routes of COVID‐19 into the body as shown in Figure 3.

The host immune response to SARS‐CoV‐2 seems to play a key role in the pathogenesis and clinical manifestations of the disease. Severe acute respiratory syndrome coronavirus not only activates the antiviral immune response, but also causes an unrestrained inflammatory response characterized by the pronounced release of pro‐inflammatory cytokines in patients with severe COVID‐19, resulting in lymphopenia and lymphocytes. However, it is the abnormalities of granulocytes and monocytes. These immune abnormalities caused by SARS‐CoV‐2 may lead to microbial infections, septic shock and severe multiple organ dysfunction (Zhu et al., 2020). For that reason, it is necessary to clarify the underlying mechanism of immune abnormality in COVID‐19 patients to guide the clinical management of the disease. In addition, reasonable treatment of the immune response of SARS‐CoV‐2 (including enhancing antiviral immunity while suppressing systemic inflammation) may be the key to successful treatment (Lin et al., 2020).

Patients with COVID‐19 and hypertension or diabetes are more probable to develop serious diseases. Delineating the mechanisms by which these chronic diseases lead to disease deterioration and a better understanding of the escape mechanism of SARS‐COV‐2 may provide clues for the clinical treatment of severe cases (Sanders et al., 2020).

In the new era, the food industry and food supply chain should solve four important issues. First, as consumers want to protect themselves and their immune systems by adopting healthier diets, the availability of bioactive ingredients in foods and functional foods become critical as the demand for these products may increase. Secondly, in order to prevent the virus from spreading among producers, retailers and consumers, food safety is an important issue. Third, food security problems have arisen because one billion people are locked in houses. Last but not least, the sustainability of the food system during the pandemic is another issue that the sector should address in order to limit related crises in the future (Galanakis, 2020).

The previous study indicates that the immune system is directly related to viral diseases patients. The decrease in peripheral T cell subsets is an exceptional characteristic in patients suffering with severe acute respiratory syndrome (SARS‐COV‐2) (Li et al., 2004). In patients recovered from this disease, a prompt reinstatement of peripheral T cell subsets is identified. Thus, peripheral T cell number can assist as a precise diagnostic tool for SARS‐COV‐2 (Li et al., 2004). The comparable phenomenon was also described in another study, in which the immune system was proved to be diminished during SARS‐COV‐2 (Cui et al., 2003). With the extrication of the association between immune responses and COVID‐19, immune characteristics are now being documented as potential biomarkers for disease progression as well as potential therapeutic targets for COVID‐19 (Perico et al., 2020).

The foods that originated from plants are intended to be used to improve the immunity of groups of people belonging to all ages against COVID‐19 (Arshad et al., 2020). It withdraws perceptions about the properties of bioactive ingredients present in foods and herbs for the provision of the immune system of humans against infections before deliberating the possibility of transmission of COVID‐19 through the food chain. It also highlights the food security issues globally arising from the fact that one‐third of the world's population is on lockdown. Finally, it emphasizes the prominence of sustainability in the food chain in order to avoid or reduce the incidence of food and health crises in the future (Galanakis, 2020).

### 4.2 Fiber reduces the risk of constipation

Constipation is a condition when fecal material moves through the colon very slowly (Sinclair, 2011). The fluid in the stool is absorbed back into our body, stool becomes dry and hard, which makes stool difficult to pass through the colon. Inadequate sleep, anxiety, poor nutrition, emotional stress, age and limited exercise are the possible causes of constipation. Various other diseases are also responsible for constipation, associated with sudden weight loss, fatigue, pain, bloody stool and changes in bowel habits (Costilla & Foxx‐Orenstein, 2014).

Fiber is a plant‐based food which is not digested in the human digestive system (García Ochoa et al., 2008). The two types of the fiber are soluble and insoluble fibers (Wang et al., 2002). Soluble fiber helps to enhance the bulk form of the stool. Apples, bananas, beans, oats and barley are good sources of soluble fiber. Insoluble fiber helps to enhance the transit of food through the digestive system which prevents constipation. Whole grains, wheat bran, legumes and vegetables are good sources of insoluble fiber. Some foods contain both soluble and insoluble fibers (Fernández‐López et al., 2009; Vitaglione et al., 2008).

The soluble fiber makes the stool bulky (McRorie & McKeown, 2017). Apples, oats, beans, barley and bananas are included in the foods which are rich in soluble fiber. The insoluble fiber helps to enhance the resistance of food in the digestive system and helps to prevent constipation. The dietary fiber present in food increases the weight and size of the stool and softens it. The bulky formed stool passes easily and lessens the risks of constipation. In the case of loose and watery stool, fiber is consumed to solidify the stool because the fiber absorbs water and maintains the bulky form of the stool (Forootan et al., 2018). Fiber helps to maintain bowel health. Fibers present in cereal have a cell wall that resists digestion and retains water in the cellular structures of fiber. Wheat bran acts as an effective natural laxative (McDougall et al., 1996).

Fresh fruits, vegetables and legumes including lentils and beans should be consumed. The fiber present in legumes and citrus fruits helps to stimulate the growth of colonic flora, increase the weight of the stool and enhance the number of bacteria in the stool. The growth of bacteria present in the colon enhances the health of the intestines. Adults should consume water‐rich foods like vegetables and fruits which are rich in fiber and help against constipation. They should be consumed according to the daily recommendation. Some studies have shown that there is a relationship between lifestyle, nutritional status and body mass index (BMI) (Karabudak et al., 2019).

Total dietary fiber has physiological effects which increase the movement in the intestine. The fiber in food accelerates the functions of the intestine by reducing the transit time of stool and its softening (Weber et al., 2014). During clinical practice, the initial management of primary constipation is done with the increased intake of fiber (Barichella et al., 2016). According to Western‐style societies, in less developed countries the main cause of constipation is the low intake of fiber. Many societies like Western Africa have made a guideline that average dietary fiber intake should include 35g of insoluble fiber, to ease the bowel movement (Sura & Christie, 2014).

An adequate amount of fiber intake softens the stool and then decreases the risk of constipation. According to previous data, the rate of constipation was higher in people whose diet was deficient in fiber (Beleli et al., 2015; Bunn et al., 2015). An adequate amount of fluid should be taken to normalize the functions of the bowels. Additionally, the increased intake of fluid can increase the efficacy of fiber‐rich foods (Sura & Christie, 2014). The nutritional status and body weight affect constipation, which is a common problem in obese patients (Pashankar & Loening‐Baucke, 2005).

According to previous literature, there is a direct relationship between an individual's constipation level and intake of dietary fiber (Kranz et al., 2012). While, according to limited studies, there is also a relationship between nutritional status, fluid intake, body mass index (BMI) and lifestyle (Misra et al., 2006).

The study was conducted to investigate the relationship between lifestyle, body weight and constipation in young healthy adults. The health of the digestive system plays an important role in our active everyday life like being free from diarrhea, bowel syndrome, bloating, abdominal pain, inflammatory bowel disease, flatulence and constipation. Dietary fibers are used to maintain the proper health of the digestive system, but there are not seen any comprehensive solutions for the health maintenance of the digestive system (Forootan et al., 2018).

Another previous review focused on summarizing the health effects of guar fiber on the digestive system. While guar fiber has been emerged to be credible for such solutions (Noack et al., 2013). According to studies, a regular intake of 5–10 g/d of guar fiber is beneficial for treating the diseases associated with the digestive system. Guar fiber is a natural fiber. It is beneficial for the protection and promotion of the health of the digestive system either separately or in combination with probiotics (Rao & Quartarone, 2019).

In children, constipation depends upon several symptoms which are likely to persist till adulthood. Whole grains and dietary fiber are used as the treatment of constipation during childhood. According to the recommendations, the fiber intake by children varies generally among the organizations. The increased intake of dietary fiber and whole grains can relieve constipation during childhood, while some additional trials are necessary for the normal recommendations (Stewart & Schroeder, 2013).

### 4.3 Colorectal cancer and fibers

Dietary fibers belong to a group of heterogeneous compounds which may vary in their biological effects. High‐fiber diet intake is responsible for reduction in the risk of colorectal cancer. The researchers assay that the fiber from whole grains and cereals can reduce the risk of colorectal cancer. Fiber can reduce the risks of colon cancer due to lessen transit time of the gastrointestinal and increasing the stool bulk and fermentation of volatile fatty acids (Hullings et al., 2020).

Fiber‐rich food lowers the risk of hemorrhoids and small pouches in the human colon. Different studies have shown that fiber‐rich diet lessens the risk of colorectal cancer. Some of the fibers are fermented in the colon (Alonso‐Coello et al., 2006).

#### 4.3.1 Normalizes bowel movements

The dietary fiber present in the diet increases the weight and size of the stool and softens it. Bulky stool can be passed easily and decreases the chances of constipation. In the case of loose and watery stool, fiber‐rich food should be consumed which solidify the stool by absorbing water and adding bulk to the stool (Nagarajan et al., 2015).

#### 4.3.2 Helps maintain bowel health

Diet rich in fiber can reduce the chances of developing hemorrhoids and small pouches in human colon. According to different studies, high‐fiber diet lowers the risk of colorectal cancer. Some of the fiber is fermented in the colon (Alonso‐Coello et al., 2006).

Dietary fiber plays a major role in the regulation of normal functions of intestines in the maintenance of healthy intestinal mucosa (Montagne et al., 2003). Some evidence has shown that the diet deficient in fiber predisposes to colon carcinogenesis (Zhu et al., 2011). The complication is that there is not any recommended level of fiber intake till now. The physicians advice their patients to consume moderate diet containing fruits, vegetables and whole grains (Demark‐Wahnefried et al., 2008). This provided a varied source of fiber‐rich foods, and their consumption optimizes the transit of the intestines depending upon the needs of every individual. Fiber supplements have been shown to be effective against colon (Klurfeld et al., 2018). Study suggested that diet rich in fiber like whole grain foods instead of fat‐rich foods can reduce the colon cancer. The results showed that diet rich in fiber like whole grain foods should be consumed instead of fat‐rich foods (Larsson et al., 2005).

Studies have shown that there is a reduced risk of colon cancer when people eat diet high in total fiber and certain whole grain foods instead of high fat foods (Kyrø et al., 2013). The animal models have shown that the dietary fiber has inhibitory effects on neoplasms depending on the nature and source of fiber (Young et al., 2005). Wheat bran inhibits the colon tumor more consistently than other types of fibers like oat and corn bran due to the presence of phytic acid which inhibits colon carcinogenesis. Human studies have revealed that the dietary fiber has modifying effect on bacterial enzymes which are involved in the production of colon tumors depending upon the fiber consumption (Holscher, 2017). Wheat bran significantly decreases the level of tumor promoters present in the colon which is not done by oat or corn bran (Zhu et al., 2011).

### 4.4 Reduce the blood sugar (diabetes) and cholesterol level

The soluble fiber dissolves in the human stomach and forms a jelly and gooey fluid, and the insoluble fiber stays as such in the human digestive system. Soluble and insoluble fibers both help with digestion, while soluble fiber has some other advantages also. Soluble fiber‐rich foods are slowly digested than processed and refined foods like cakes, cookies and white bread. Dietary fiber affects the activity of enzymes present in the intestine and then regulates the metabolic activity of the body. It lessens the speed of digestion which relieves the release of glucose and control the blood sugar up to an optimal level. Sugar is absorbed from food by the human body very slightly which increases blood sugar level slowly and prevents the human body from a sudden increase in blood sugar level (Brennan & Tudorica, 2008).

According to many studies, high carbohydrates in the diet have adverse effects but can be minimized by increasing the content of carbohydrates and fiber simultaneously in diabetic patients’ diet (Czyzewska‐Majchrzak et al., 2014). Particularly, many studies demonstrated that a diet rich in carbohydrates and fiber can enhance the glucose control of blood and lessen the cholesterol level of plasma by comparing the diet low in carbohydrates and fibers for diabetic patients. Additionally, a diet rich in fiber and carbohydrates cannot increase insulin and triglyceride concentration in plasma (Reynolds et al., 2019).

The ability of dietary fibers to retard nutrient absorption and food digestion has a very important impact on the metabolism of carbohydrates and lipids. The beneficial effects of fiber‐rich foods are also exerted by foods not rich in fiber. The fiber content and physical form of food influence the accessibility of nutrients by the activity of enzymes, which delay the digestion and absorption processes. Identification of foods with low glycemic response helps to enlarge the number of foods mainly for diabetic patients. A diet deficient in saturated fat and cholesterol is mainly recommended to diabetic patients to control cardiovascular diseases. A diet rich in saturated fat and cholesterol should be replaced by a diet rich in fiber and unsaturated fat and consumed by diabetic patients. High intake of dietary fiber, mainly soluble fiber can improve glycemic control, lower the lipid concentrations of plasma and lessen hyperinsulinemia in diabetic patients of type 2 (Guo et al., 2018).

Fiber can reduce glucose levels in the blood. Fiber inhibits the glucose passage from intestinal walls to bloodstream and controls the blood glucose levels. According to previous studies, there is a significant relationship between fiber intake and blood glucose level in diabetic patients. Study suggested that there is a link between fiber intake and glucose level of the blood. The results showed that low fiber intake increases the glucose level of blood (Bertalina & Rajiani, 2018).

Psyllium is a dietary fiber used to lower blood cholesterol and blood sugar (Ziai et al., 2005). Soluble fiber lessens the absorption of cholesterol into the bloodstream. The intake of soluble fiber lessens the concentration of LDL cholesterol (Jesch & Carr, 2017). Psyllium is a fiber which reduces the concentration of LDL cholesterol in the bloodstream. Psyllium is a type of fiber which is found in psyllium husk (Slavin, 2013).

The previous study focused on the use of seaweed to prepare the dietary fiber by microbial transformation of *Lactobacillus rhamnosus* and *Lactobacillus plantarum* (Gupta et al., 2011). Studies have shown that the concentration of soluble dietary fiber increases after the action of mixed lactic acid bacteria and the rate of incrimination was 21.1%. After fermentation, the inhibition rate of α‐amylase and α‐glucosidase increased by 25.9% and 16.6% respectively. While, after fermentation, seaweed increases the concentration of soluble dietary fiber and increases the inhibition of enzymes responsible for digestion. Dietary fibers present in seaweed are responsible for hypoglycemic effects (Zhou et al., 2019).

#### 4.4.1 Fiber lowers cholesterol

Soluble fiber is also present in brussels sprouts, pears, apples and kidney beans. These soluble fibers reduce the absorption of cholesterol in the bloodstream (Gunness & Gidley, 2010). The soluble fiber present in oatmeal reduces the concentration of low‐density lipoprotein cholesterol which is also called bad cholesterol (Othman et al., 2011).

The soluble fiber lowers the cholesterol level by binding it to the small intestine. During passing through the small intestine, the fiber attaches with the cholesterol particles and prevents them from entering the bloodstream and then excreting them from the body through feces (Gunness & Gidley, 2010).

The main biological mechanisms of soluble dietary fibers used to lower the cholesterol are excretion of bile salts by inhibiting their absorption in the small intestine, lowering of stimulation of insulin for hepatic cholesterol synthesis which is the result of reduced glycemic response and effects of soluble dietary fiber. The studies suggest that the micelles of bile salts bind with soluble dietary fibers which inhibit their reabsorption. While different studies have been conducted to research the glycemic effect of soluble dietary fibers, which suggested that the micelles of bile salts can interact with soluble dietary fibers which inhibit their absorption (Tosh & Bordenave, 2020). The three physiochemical mechanisms are proposed together with the suggestion of in vitro experiments to test them (Gunness & Gidley, 2010).

Previous study shown that there is a positive relationship between diet having soluble dietary fibers (guar gum, psyllium, pectin and β‐glucan) and lowering of cardiovascular diseases and cholesterol in blood serum (Theuwissen & Mensink, 2008).

### 4.5 Hypertension

Fibers consist of indigestible parts of plants which are not digested in our digestive system. Fibers are mainly carbohydrates, and their role is to keep the digestive system healthy (Mudgil & Barak, 2013). The fiber‐rich diet is associated with significant reduction of both systolic and diastolic blood pressure in the people with high blood pressure (Keenan et al., 2002). Studies have shown that fiber‐rich foods have many other health benefits like reduction in blood pressure and inflammation (Papanikolaou & Fulgoni, 2008). Soluble fiber can slow down the absorption of sugar and improve the blood glucose level in diabetic patients (Abutair et al., 2016; Aleixandre & Miguel, 2016).

The main factors associated with hypertension and blood pressure are increased population trends of obesity, overweight and aging. The cases of hypertension can be controlled by changing the lifestyle factors like alcohol consumption, poor dietary patterns, sedentary lifestyles, smoking and excess energy intake. Studies have shown that increased intake of fiber up to 7–15 g/d above the normal level is associated with reduction of hypertension and blood pressure. Meta‐analysis trials have shown that increased intake of fiber is effective for lowering the blood pressure in elders and obese and elevated hypertension and blood pressure in younger, leaner and normotensives. Soluble fiber including beta‐glucan is effective in lowering the blood pressure more than the insoluble fiber obtained from wheat bran. The mechanism of fiber to lower the blood pressure included the reduction of risks of weight gain and obesity, improving the vascular health by lowering the LDL cholesterol and lowering the inflammation via colonic microbiota (Dreher, 2018a, 2018b).

The role of fiber in preventing heart disease is related to its ability to lower cholesterol and blood pressure. Soluble fiber present in oat grain including beta‐glucan is responsible for the reduction of cholesterol of plasma. According to many studies, dietary fiber may improve insulin resistance, weight loss and glycemia. Study was conducted on hypertensive rats in which dietary fiber affects the arterial blood pressure. Besides that, some types of fiber improve the arterial blood pressure. Besides that, some types of fiber improve the arterial blood pressure, and it has been proved by the studies on hypertensive and prehypertensive subjects. This study reviewed the researches done to analyze the antihypertensive effects of dietary fiber and their effects on the cardiovascular diseases (Aleixandre & Miguel, 2016).

## 5 CONCLUSION

It is concluded that plant‐based foods composed of rich nutritive components. These nutritive components play a separate role in human health. From all of these components, fiber is a nondigestible polysaccharide available in plant food like vegetables, fruits, legumes and cereals. Pectin, beta‐glucan, mucilage, psyllium, resistant starch and inulin are present in soluble form, whereas cellulose, hemicellulose and lignin are in insoluble form in different varieties of plant food. The fiber vital function is to boost immunity that aids human from viral diseases. The COVID‐19 is viral disease spread overall the world. However, the attack of coronavirus are detected lower in those peoples who have strong immune systems. Fiber played a medicinal role to protect the human body from acute or chronic diseases. Fiber can help to reduce the chances of hypertension and hyperglycemia because a high‐fiber diet is the ability to lower cholesterol, blood pressure and blood sugar. It boosts the gut microbes that protect GIT and prevent constipation because it absorbs water and adds bulk to stool.

## ACKNOWLEDGEMENT

The authors are highly thankful to Government College University Faisalabad, Pakistan for providing the facilities.

## Data Availability

No data available because it is a review article.
